# Literary Notes

**Published:** 1882-10

**Authors:** 


Literary Notes.—Professor Th. Leisering, of the Royal Veterinary School at Dresden, has published a fifth and enlarged edition of his work on the horse's foot. It contains one hundred and fifty-nine wood-cuts.

Dr. O. Eversbusch, of the Munich Veterinary School, has written a short clinical work on diseases of the eye.

Prof. Putz, of Halle, has written a book upon the epidemic and endemic diseases of our domestic animals.

Dr. P. Adam, of Zweibrucken, is publishing a series of monographs on the anatomy, physiology and diseases of the horse.

An excellent and much-needed work upon diseases of our domestic fowls has just appeared from the pen of Prof. F. A. Zurn, of Leipzig.

